# Lysosomal Acid Lipase Deficiency: Genetics, Screening, and Preclinical Study

**DOI:** 10.3390/ijms232415549

**Published:** 2022-12-08

**Authors:** Ryuichi Mashima, Shuji Takada

**Affiliations:** 1 Department of Clinical Laboratory Medicine, National Center for Child Health and Development, 2-10-1 Okura, Setagaya-ku, Tokyo 157-8535, Japan; 2 Department of Systems BioMedicine, National Research Institute for Child Health and Development, 2-10-1 Okura, Setagaya-ku, Tokyo 157-8535, Japan

**Keywords:** lysosome acid lipase, screening, genetics, biochemistry

## Abstract

Lysosomal acid lipase (LAL) is a lysosomal enzyme essential for the degradation of cholesteryl esters through the endocytic pathway. Deficiency of the LAL enzyme encoded by the *LIPA* gene leads to LAL deficiency (LAL-D) (OMIM 278000), one of the lysosomal storage disorders involving 50–60 genes. Among the two disease subtypes, the severe disease subtype of LAL-D is known as Wolman disease, with typical manifestations involving hepatomegaly, splenomegaly, vomiting, diarrhea, and hematopoietic abnormalities, such as anemia. In contrast, the mild disease subtype of this disorder is known as cholesteryl ester storage disease, with hypercholesterolemia, hypertriglyceridemia, and high-density lipoprotein disappearance. The prevalence of LAL-D is rare, but several treatment options, including enzyme replacement therapy, are available. Accordingly, a number of screening methodologies have been developed for this disorder. This review summarizes the current discussion on LAL-D, covering genetics, screening, and the tertiary structure of human LAL enzyme and preclinical study for the future development of a novel therapy.

## 1. Introduction

Lysosomal acid lipase deficiency (LAL-D) (OMIM 278000) is a lysosomal storage disorder (LSD) characterized by an accumulation of cholesteryl esters and triglycerides in the lysosome [1,2,3,4,5,6]. A severe disease subtype of LAL-D is called Wolman disease, manifesting through various symptoms, such as hepatomegaly, splenomegaly, vomiting, diarrhea, adrenal calcification, and hematopoietic disorders, including anemia. Patients typically fail to survive beyond two years [7,8,9]. A mild disease subtype of LAL-D is called cholesteryl ester storage disease (CESD). In this case, patients suffer from hypercholesterolemia, hypertriglyceridemia, high-density lipoprotein (HDL) deficiency, and dysregulated lipid deposition [7]. Patients affected with CESD survive into adulthood. The current understanding of LAL-D is that this disorder is caused by a pathogenic mutation of the *LIPA* gene on chromosome 10q23.2-q23.3 with a very wide spectrum of clinical manifestations rather than two separate disorders. These manifestations are associated with no to low enzyme activity of LAL [10]. An elevation of enzyme activity of transaminases in the liver, such as ALT and AST, is considered a surrogate marker. Although changes in these alone may not provide a conclusive diagnosis, this evidence helps to make an accurate diagnosis. Biochemically, LAL is capable of degrading lipids such as cholesteryl esters, triglycerides, and retinyl esters. To assist diagnosis of LAL-D, an identification of adrenal calcification using imaging diagnosis with CT was used [11,12].

There are several treatment options for LAL-D. One is enzyme replacement therapy (ERT), in which patients receive a recombinant enzyme agent biweekly. The intravenously infused enzyme agent is then taken up by cells at or near the site of the disorder. Biochemically, the enzyme targets the lysosome in a mannose-6-phosphate-dependent manner. This mechanism is called a cross correction, a commonly observed biochemical process of the cellular intake of LSD enzymes [13,14]. An enzyme agent for LAL-D was approved by the FDA in 2015 [15,16]. A combination therapy of ERT and hematopoietic stem cell transplantation (HSCT) can be considered depending on the case [17]. In LAL-D, dietary substrate reduction may be performed combined with ERT or HSCT [17] by eliminating cholesteryl esters and triglycerides from the diet.

This Review aims to summarize recently published data within the last 5 years. In particular, great progress has been made in: (i) the understanding of the genetics of pathogenesis of LAL-D based on screening by both genetic and biochemical techniques; (ii) the tertiary structure of LIPA protein; (iii) the phenotype of animals of *Lipa*-deficient mice. Although these data are studied in each specific research area in an individually distinct manner, integration of them would enhance the benefit for the affected individuals with the possible development of a novel therapy with evidence. Specifically, for example, an accumulation of genetic data increases the accuracy of identification of a Wolman patient who needs to commence an immediate initiation of therapy. An understanding of LIPA enzyme structure would provide a wider chance of drug development. The results of the animal study would provide a variety of phenotypes in LAL-D-deficient patients, including both Wolman and CESD. Note that important references prior to this period were also included in order to help understand the historical background of LAL-D research.

## 2. Genetics

### 2.1. Prevalence

LAL-D is a rare disease [18]. A meta-analysis study estimated that the frequency of LAL-D was 1 in 177,000 [18]. In this study, the frequency of LAL-D was reported to be low among Finnish, East Asian, and South Asian patients and high among non-Finnish European patients. The number of mutations known is increasing; there are currently over 130 variants [1]. Overall, 50–60% of pathogenic alleles are linked to the E8SJM mutation. Except for this mutation, most mutations occur by chance. In other words, there is no evidence that recombination, large deletion, or other translocation occurs in the *LIPA* gene at a high frequency.

### 2.2. E8SJM (c.G894A; p.delS275_Q298)

There is a characterized pathogenic mutation in exon 8 (c.G894A; p.delS275_Q298) (Table 1) [1,16,19]. This is a mutation at the splicing joint of exon 8, leading to its splicing abnormality. As a result, the mutated LAL protein without enzyme activity, lacking a short peptide with 24 amino acids, was generated as a mature protein. In the homozygote of the mutant E8SJM, small residual enzyme activity is reported, which occurs due to the generation of wild-type enzymes by an incorrect splicing of the pathogenic genome [1].

### 2.3. Single Nucleotide Polymorphism (SNP)

There are two SNPs on exon 2: rs1051338 (c.A46C; p.T16P) and rs1051339 (c.G67A; p.G23R) (Table 1). The mutation of the *LIPA* gene involving these two SNPs causes alterations of amino acid sequences on the signal peptide of the *LIPA* transcript, leading to impaired lysosomal targeting and instability of a wild-type LAL enzyme [23]. Consistently, there is no evidence of pathogenicity in these genomic alterations.

## 3. Diagnosis and Screening

### 3.1. Biochemical Assay

LAL is a lysosomal enzyme that is active in acidic pH. Due to the presence of multiple isozymes of lipase in vivo [21,22], selective quantification of LAL enzyme activity from other lipases, such as lipoprotein lipase, is expected. Hamilton et al. developed a LAL assay using the LAL-specific inhibitor Lalistat-2 in dried blood spot (DBS) with 4-methylumbelliferone (4-MU)-based fluorometric quantification [10,29]. In this assay, the LAL enzyme activity was calculated as a Lalistat-2-inhibitable enzyme assay. This protocol is widely used in clinical laboratories [30,31]. Several populations of hepatic disorders, such as fatty liver, cirrhosis, and dyslipidemia, were screened by high-risk screening. Subsequently, an LAL-specific substratewas developed [32]. This substrate was successfully applied to LC-MS/MS [32,33].

### 3.2. Surrogate Markers

Surrogate markers are defined as biomarkers that change their concentration under pathophysiological conditions but are not directly associated with the cause of the disease. In LAL-D, elevated liver enzymes, such as ALT and AST, act as surrogate markers [15]. An elevation in cholesterol is associated with LAL-D [17]. Because elevation of these alone does not provide any conclusive results, an enzyme assay for LAL-D should be performed for diagnosis. Consistent with cholesterol accumulation, recent studies suggest that cholesterol 3β,5α,6β-triol could also be a reliable biomarker for LAL-D [34,35].

### 3.3. High-Risk Screening

High-risk screening is used to identify affected individuals in a relatively small population according to a range of criteria, such as age, ethnicity, or specific disease (e.g., familial hypercholesterolemia) (Table 2 and Table 3). The advantage of high-risk screening is that it increases the chance of patient identification. In such cases, the study was designed to identify at least one patient in the testing population. Therefore, the choice of population is a key determinant of the study design. Although previous studies preferred to opt for a biochemical assay, possibly due to its affordability, some current studies prefer a genetic assay, possibly due to a lack of quantitative biomarkers for diagnosis.

#### 3.3.1. Genetic High-Risk Screening

To understand the identification of LAL-D-affected individuals, several high-risk screening studies have been performed (Table 2). In cohort-based studies, an earlier study identified a high E8SJM mutation prevalence in the German population [18,19,27]. Due to the association between lipidemia and LAL-D, subsequent studies have been designed to identify *LIPA* variants in dyslipidemia. For example, a U.K. study demonstrated that a population with familial hypercholesterolemia (FH) and no pathogenic mutations of *LDLR*, *ApoB*, and *Pcsk9* contained LAL-D-affected patients [23,24].

**Table 2:** High-risk screening for LAL-D using genetic assay.

Method	Year	Country	Author	Population	n			LDLR	ApoB	Pcsk9	APOE	LDLRAP	ABCG5	ABCG8	LIPA	STAP1	Others	Ref.
					Total	Children	Adults											
NGS+Sanger	2022	Slovenia	Sustar, U	FH	669	669	0	189 (LDLR+ApoB+Pcsk9)			NR	NR	NR	NR	3	NR		[20]
NGS	2021	Netherlands	Reeskamp, LF	FH	1528	0	1528	227 (LDLR+ApoB+Pcsk9)			0	0	0	2	0	NR		[36]
NGS+Sanger	2021	Russia	Miroshnikova, VV	Suspected FH	59	28	31	24	5	1	NR	0	3	2 (ApoB+ABCG8)	1	NR		[22]
NGS+Sanger	2020	Brazil	de Paiva Silvino, JP	FH	143 (a)	Combined		5	1	1	1	0	0	0	0	0		[37]
NGS	2020	Argentina	Corral, P	FH	246	0	246	12	1 (ApoB+ApoE)	NR	1 (ApoB+ApoE)	NR	NR	NR	1	NR		[38]
NGS+Sanger+TaqMan	2019	Gran Canaria (Spain)	Sánchez-Hernández, RM	FH	70	0	70	41	1	1	0	0	NR	NR	1	0		[39]
NGS+Sanger	2018	Slovenia	Groselj, U	FH	170	170	0	49	27	0	NR	NR	NR	NR	1	NR		[40]
NGS	2018	Slovenia	Corral, P	Hypercholesterolemia	69	0	69	20	3	0	2	0	7 (ABCG5+ABCG8)		2	NR	CYP27A1(3), LIPC(1), LIPG(1), LPL(0), and SCARB1(1)	[41]
Sanger	2020	Portugal	Mariano, C	FH	731	311	420	282 (LDLR+ApoB+Pcsk9)			NR	NR	NR	NR	3	NR		[42]
Sanger+TaqMan	2019	U.K.	Ashfield-Watt, P	FH	663	0	663	NR	NR	NR	NR	NR	NR	NR	3	NR		[23]
Sanger	2017	Portugal	Chora, JR	FH +dyslipidemia (b)	750	193	557	NR	NR	NR	NR	NR	NR	NR	4	NR		[43]
Sanger	2016	Netherlands	Sjouke, B	FH	276	63	213	Excluded	Excluded	NR	NR	NR	NR	NR	6	NR		[24]

NR, not reported; FH, Familial hypercholesterolemia. (a) 32 index cases and 111 relatives; (b) FH (*n* = 492) + dyslipidemia and/or liver steatosis (*n* = 258). Gene abbreviation: ABCG5, ATP binding cassette subfamily G member 5; ABCG8, ATP binding cassette subfamily G member 8; ApoB, apolipoprotein B; ApoE, apolipoprotein E; CYP, cytochrome; LDLR, low-density lipoprotein receptor; LDLRAP, low-density lipoprotein receptor accessory protein; LIPA, lipase A, lysosomal acid; LIPC, lipase C, hepatic type; LPL, lipoprotein lipase; Pcsk9, proprotein convertase subtilisin kexin 9; SCARB, scavenger receptor class B member; STAP1, signal transducing adaptor family member 1.

Sjouke et al. reported the results of high-risk screening of a population with phenotypically diagnosed FH subjects (*n* = 276; 213 adults and 63 children; 105 males (38.0%)) [24]. In this study, the aforementioned major variant and SNPs such as E8SJM (2 heterozygotes), c.A46C (p.T16P; rs1051338; 101 heterozygotes and 12 homozygotes), and c.G67A (p.G23R; rs1051339; 43 heterozygotes and 3 homozygotes) were reported. In addition, some minor SNPs such as c.683 T>C (p.F228S; rs2228159; 2 heterozygotes; possibly pathogenic); c.913 T>A (p.F305I; not registered; 1 heterozygote; possibly pathogenic); c.966+3 A>T (putative primary structure not reported; rs201242614; pathogenic) were identified (Table 1 and Table 2).

Sustar et al. reported a genetic high-risk screening as part of Slovenian universal FH screening [20]. The authors screened LAL-D as a secondary condition of FH-related hyperlipidemia. Among the 669 children with dyslipidemia, they identified three affected individuals with a homozygous E8SJM (c.G894A) mutation. The authors suggest a beneficial role of genetic screening in children, as it can provide earlier detection of individuals affected by rare dyslipidemia at the early stage of their lives. To distinguish LAL-D from FH, the elevated activity of transaminases, such as AST and ALT, can be used as a surrogate biomarker.

Miroshnikova et al. reported high-risk screening of FH by the genetic method in Russia [22]. The authors recruited 59 unrelated patients (*n* = 31 adults; *n* = 28 children/adolescents). FH-associated genetic mutation was detected in 58% (*n* = 18) of the adults and 89% (*n* = 25) of the children. As a result, they identified multiple *LDLR* variants with a small number of *ApoB* variants. Additionally, one case of the *LIPA* variant was identified in their study.

Corral et al. reported a high-risk screening of FH detection programs in Argentina (da Vinci Study) [38]. Among 246 hypercholesterolemic patients, 21 individuals with definite diagnoses were selected. This sample was genetically analyzed using NGS with an extended panel of 23 dyslipidemia-related genes. Among the 21 patients, more than half (*n* = 12) had a variation in *LDLR*. They also identified a *LIPA* variant. Six individuals (28%) had no apparent mutation in the NGS panel, which raised the possibility that novel genes not listed in the NGS panel could be involved.

#### 3.3.2. Biochemical High-Risk Screening

Tebani et al. reported the results of a large-scale clinical screening of LAL-D in a French population (Table 3) [44]. In their study, the authors selected a population for clinical screening based on unexplained hepatomegaly, an increase in transaminase activity, disturbed serum lipid profile with or without splenomegaly, gastrointestinal dysfunction, or hepatic microvascular steatosis/fibrosis/cirrhosis. The study was performed between 2015 and 2019, and the average ages of men (*n* = 2494) and women (*n* = 1690) in the sample were 44.88 ± 20.49 and 42.78 ± 22.56 years, respectively. The study also included children under two years of age (*n* = 157), both male and female.

The authors reported a mean LAL activity of 0.89 ± 0.54 nmol/punch/h in the DBS samples [44]. Overall, the study identified six Wolman- and 13 CESD-affected individuals. Wolman patients involved 2 males and 4 females with an average age at diagnosis of 3.12 ± 4.08 months. CESD patients involved 4 males and 9 females with an average age at diagnosis of 22.42 ± 18.23 years. Wolman-affected individuals were only detected in children, and CESD-affected individuals were in adults, respectively. The detected mutations included nonsense, frame shift, and splicing variants; the spectra of the pathogenic mutations in Wolman and CESD were distinct. Notably, the common variant E8SJM/c.G894A, which accounted for 12 out of 34 mutated alleles, was found only in the CESD-affected individuals. Note that no difference was observed in LAL activity between the Wolman- and CESD-affected individuals.

Mayanskiy et al. reported the LAL activity of DBS in a Russian population [45]. In their study, the authors researched a sample of 537 LAL-D-suspected individuals undergoing clinical screening between June 2016 and July 2018. The sample included 239 children (44.5%) under five years of age. The genetic analysis identified affected (*n* = 6) individuals and carriers. To obtain further insight into the prevalence of E8SJM/c.G894A, a merged population, including the six samples identified in this study and 12 previously diagnosed samples, were examined. In this pool, the authors identified 16 individuals who had this mutation with six homozygotes. An inverse correlation of LAL-D enzyme activity among patients under 10–15 years of age was reported.

Originally, Hamilton et al. reported the inclusion of Lalistat-2 in DBS to quantify LAL enzyme activity [10,29]. However, Mayanskiy et al. reported a kinetic assay of the LAL enzyme without Lalistat-2, showing 100% sensitivity of LAL-D-affected individuals (*n* = 6) and near-complete sensitivity of non-LAL-D individuals (103 out of 105) [45]. As long as assay specificity is guaranteed and assay equipment is available, the latter assay can be useful. Notably, under this assay condition, the LAL-D-affected individuals showed approximately 20% lower non-LAL-derived basal activity, implying attenuation of these activities in LAL-D patients.

**Table 3:** High-risk screening for LAL-D enzyme using DBS.

Method	Year	Country	Author	Population	n			Affected		Ref.
					Total	Children	Adult	Wolman	CESD	
4MU	2021	France	Tebani, A	Clinical screening	4174	157	4017	6	13	[44]
4MU	2019	Russia	Mayanskiy, N	Clinical screening	537	239	298	6 positives		[45]
4MU	2018	U.K.	Reynolds, TM	↓HDL; ↑ALT	1825	Combined		0	0	[46]
4MU	2017	Italy	Tovoli, F	NAFLD	159	0	159	0	0	[47]
4MU	2017	Italy	Vespasiani-Gentilucci U	Healthy subjects	172	0	172	0	0	[29]
4MU	2016	Italy	Vespasiani-Gentilucci, U	Cirrhosis + Healthy controls	252	0	252	0	0	[48]
4MU	2016	Italy	Selvakakumar, PKC	Children with NAFLD	168	168	0	0	0	[49]
4MU	2016	Israel	Shteyer, E	Cirrhosis without known etiology	22	Combined		0	0	[50]
4MU	2015	Italy	Baratta, F	Adult NAFLD	240	0	240	0	0	[51]
4MU	2014	Japan	Dairaku T	CESD + Normal control	65	Not described		0	7	[28]

ALT, alanine transaminase; HDL, high-density lipoprotein; NAFLD, nonalcoholic fatty liver disease.

### 3.4. Newborn Screening

Newborn screening is used to identify disease-affected individuals at an early age after birth [52]. Such screening was initially proposed by Dr. Guthrie in the 1960s for the treatment of phenylketonuria [53]. The earlier assays used microbial procedures; however, the current assays use modern instruments for the quantification of biomarkers, including metabolites and enzyme activity. Today, newborn screening is implemented with a variety of metabolic disorders, including LSDs and immune and neurological disorders [54,55,56], which necessitates a robust screening methodology and disease-specific therapy. In addition, the prevalence is another factor to be considered.

A type of blood formulation called DBS has been used for newborn screening. The application of DBS for quantification includes amino acids and acylcarnitines [53], LSD enzymes [52,54,56], T cell receptor excision circle (TREC), and survival motor neuron (SMN) [57,58]. As the target biomarker (metabolites, enzyme activity, genome, etc.) is stable under the specified transportation conditions, the usefulness of DBS for newborn screening (NBS) has been widely appreciated. As listed in Table 3, the LAL enzyme remains active when DBS is properly prepared. In LAL-D, no newborn screening is currently performed. Based on the success of high-risk screening in Russia [45] and France [44], newborn screening for LAL-D is increasingly demanded.

## 4. Tertiary Structure of Human LAL Protein

Until recently, the structure of the human LAL enzyme has been only postulated according to two established structures of lipases, such as human gastric lipase [59] and dog gastric lipase [60]. In general, lipases are involved in α/β hydrolase superfamily similar to serine proteases, and this superfamily has a conserved amino acid triad consisting of serine, histidine, and asparagine. These three conserved amino acids are also found in human gastric lipase and human LAL as well. Human gastric lipase has a core domain and a cap domain with a lid subdomain [59]. The lid subdomain is capable of translocating between closed and open states. Based on the similarity of the primary structure of the human LAL enzyme, it has been postulated that human LAL might have a similar structure. Homology-based modeling is a software-based strategy that allows the prediction of the pathogenesis of uncharacterized mutants for CESD and Wolman disease based on the primary structure of the human LAL enzyme [61].

Recently, Rajamohan et al. reported the crystal structure of the human LAL protein [62]. Overall, the structure of the human LAL enzyme is found to be similar to other members of the hydrolase superfamily involving human gastric lipase. Human LAL enzyme has the core domain consisting of a central β-sheet composed of eight strands and two sets of three helices on each side. The cap domain of the human LAL enzyme contains a lid structure (residues 215–244) that adapts a closed conformation. A comparison of the lid-open form and the lid-closed form of human LAL enzyme revealed that Pro214 and Gly245 act as hinges between the cap and core domain structures. A biochemical experiment deleting the 7-amino acid sequence (resides 238–244: NLCFLLC) significantly impaired LAL enzyme activity at acidic pH [62]. Based on structural data, this inactivation is caused by an impaired binding of the mutant protein and a substrate. The proper activation only occurred in wild-type enzymes at acidic pH, whereas a weak binding of the enzyme and substrate was usually observed at neutral pH.

An H274Y mutant is an identified LAL mutant identified in CESD-affected individuals [62]. The activity of the lipase enzyme in this mutant is less than 5%, but the structural rationale behind this phenomenon remains undiscovered [63]. Based on the newly reported structural data for the human LAL enzyme, His274 has been, for the first time, identified as a key amino acid interacting through hydrophobic interaction with Ser122 and Gln285 of the core domain. A molecular dynamics study revealed that this H274Y mutant had impaired flexibility of the putative lid subdomain in the LAL enzyme.

Finally, the E8SJM mutant, lacking a short sequence of 24 amino acids arising from exon skipping, has no enzyme activity [1,16,17]. According to the tertiary structure of the wild-type human LAL enzyme, this mutant lacks the abovementioned short sequence at the C-terminal region of the cap domain. In this case, the primary structures of the core domain and the lid subdomain remain intact, suggesting that a substantial alteration of the cap domain may be involved.

## 5. Phenotype of *Lipa*-Deficient Mice

Mice deficient in the *Lipa* gene have been used as the disease model of human pathology to understand its mechanism. In mice, three major phenotypes—hepatic lipid accumulation, immune modulation, and tumorigenesis—have been reported. As mentioned, abnormal lipid accumulation is a hallmark of LAL-D, including both Wolman and CESD [1]. Pancytopenia and hyper-inflammation, together with hypercytokinemia and hemophagocytic lymphohistiocytosis arising from the abnormality of immune cells, may be observed in Wolman disease [17]. Although, to a lesser extent, two cases of hepatocellular carcinoma were also reported in CESD [1]. Thus, these manifestations in humans could be recapitulated by *Lipa*-deficient mice. Less well characterized is the role of cholesterol accumulation in LAL-D and homeostatic gene expression relevant to lipid accumulation, immunity, and tumorigenesis.

### 5.1. Lipid Accumulation

Based on the existing data, the phenotype of the liver appears to be similar to that of CESD rather than Wolman (Table 4). Essentially, *Lipa*-deficient mice showed an increase in cholesteryl esters in their livers; however, the accumulation of triglycerides and retinoic acid appears to be inconclusive. According to the results of liver-specific *Lipa* deficiency, hepatic TG and RA remain unaltered in normal chow. However, an approximately 30% reduction of triglycerides in the liver in the liver-specific *Lipa*-cKO mice with a high-fat diet containing vitamin A at the age of five months [64]. These results clearly demonstrated that hepatic LAL plays a key role in CE metabolism in the liver, while hydrolysis of triglyceride and retinoic acid is mediated by uncharacterized lipase.

### 5.2. Immune Cells

In Wolman disease, impaired hematopoiesis, including anemia, is occasionally reported [1,51]. Considering that hepatomegaly is a common phenotype in humans and mice, extramedullary hematopoiesis, an alternative process of hematopoiesis other than the bone marrow, may be particularly important in humans [79]. The increasing proliferation ability of hematopoietic stem cells and granulocyte-monocyte progenitor cells of *Lipa*-deficient mice suggests an enhanced tyrosine kinase-mediated signaling pathway in these mice [69]. This observation is consistent with other immune cells in *Lipa*-deficient mice (Table 4).

Nguyen et al. reported the role of the *Lipa* gene in graft-versus-host disease (GVHD) [80]. The authors examined its role in the murine model of allogenic hematopoietic cell transplantation and found that this gene was essential for donor T cell survival, differentiation, and alloreactivity in the GVHD’s target organs but not in lymphoid organs. In the *Lipa*-deficient T cells, a preferential differentiation of T cells into Th1 and Th17 with an impaired differentiation to the regulatory T cells, both of which lead to anergic conditions in target organs, was observed, which can, at least partially, be explained by an enhanced susceptibility of the *Lipa*-deficient mice to oxidative stress. This observation was recapitulated by suppressing LAL enzyme activity by a small molecule, such as orlistat. Thus, the results demonstrated that LAL plays an important role in controlling GVHD and tumor growth after allogenic hematopoietic cell transplantation.

### 5.3. Tumorigenesis

The role of the *Lipa* gene in tumorigenesis has been studied in murine models [73,77,81]. In *Lipa*-deficient mice, transplanted melanoma grew rapidly compared to controls. The reason for this can be associated with enhanced tumor metastasis [77]. To obtain further insight into this observation, enhanced angiogenesis of endothelial cells in *Lipa*-deficient mice has been suggested [73]. Notably, there is a case study reporting cholangiocarcinoma suffered by a CESD patient with a compound heterozygote of the *LIPA* gene with c.G894A and ΔC(673–675) [82,83]. In this case, a massive accumulation of histiocytes was noted.

Liu et al. reported an anti-tumor role of the *Lipa* gene in the ER stress-mediated mechanism [84]. The authors were the first to identify a stereo-specific small molecule (ERX-41) with high binding properties to LAL protein. In their study, they showed that ERX-41 is a novel ER localization regulator through the LAL protein without modulating its enzyme activity.

## 6. Treatment

Since enzyme replacement therapy was approved in 2015 in the U.S., patients in most European and Asian countries have also had access to this agent [15]. Apart from that, dietary substrate reduction therapy is an emerging therapy that successfully reduces lipid levels in patients by lowering the lipid content in their diet when used with ERT or HSCT [17]. For CESD-affected individuals, the use of pharmacological agents, including statins and ezetimibe, has been considered [1]. Liver transplantation has also been considered [1]. Apart from these established treatment options, a novel therapy, including gene therapy using adeno-associated virus (AAV), has been examined in the murine model [85].

### 6.1. Enzyme Replacement Therapy

Several preclinical studies have been performed using *Lipa*-deficient mice (Table 5), in which the therapeutic effect of intravenously administering the purified recombinant LAL protein was studied. For example, Sun et al. reported that *Lipa*-deficient mice were treated with different levels of LAL enzyme, ranging from 0.8 to 3.2 mg/kg [86]. Under these experimental settings, the lifespan of the treated rodents was extended by 52 and 92 days at 0.8 mg/kg and 3.2 mg/kg, respectively, and the accumulation of cholesterol and triglyceride in their livers and spleens decreased. At 10 mg/kg, the treated mice showed normalized organ sizes and liver, spleen, and intestine histology. These results demonstrated that the recombinant human LAL enzyme was active and safe in the mouse model. For the development of an enzyme agent for clinical use, a rat model was used [87].

### 6.2. Gene Therapy

Lopresti et al. reported the effectiveness of AAV-mediated gene therapy in the *Lipa*-deficient model [85]. In their study, the thyroxine-biding globulin-driven *LIPA* gene was carried in AAV encapsulated with the AAV8 serotype (an established serotype that targets the liver). To mimic the nonalcoholic fatty liver disease (NAFLD) condition, an FPC diet (a high-fat diet with additional fructose, palmitate, and cholesterol) was administered. In this model, a cohort of mice (9 weeks old) was divided into two groups at week 0, followed by AAV administration at 5 × 10^11^ vector copies/mouse through a retroorbital injection. Then, these mice were further divided into two groups at week 1: one was treated with the common Western diet, and the other one was treated with the control diet. At week 16 (26 weeks old), the mice showed the same body weight in males, with an increased liver weight following the Western diet treatment. Under this treatment condition, both the activity and protein of LAL in the liver were elevated in the AAV-administered animals. Most strikingly, the authors reported a larger accumulation of Kupffer cells in the *LIPA*-administered mice with the Western diet compared to the GFP-administered control mice. This observation was reconciled by increased autophagocytosis with elevated LC3-II protein expression in the AAV-administered mice.

Lam et al. reported a therapeutic effect of rscAAVrh74-mediated *LIPA* expression [91]. In their study, the therapeutic vector was administered at post-natal day 2 (P2) (neonate) through the facial vein in a sample of young mice (P60) and through the tail vein in adult mice (P120). The elevated alanine aminotransferase (ALT) and aspartate aminotransferase (AST) enzyme activity in the *Lipa*-deficient mice was significantly decreased at P60 and P120 when the vector was administered at a dose of 8.4 × 10^13^ vector genomes (vg) per kilogram (vg/kg) intravenously. They also reported a wide distribution of the AAV vector in the liver, spleen, intestine, and lymph node at P60 and P120, while in the heart and lung, it was comparable to the mice that were administered at P2. Similarly, LAL enzyme activity was detected in the liver at P60 and P120, while it was only significantly elevated in the spleen at P120. In addition to enzyme activity, triglyceride, a major class of lipids in the liver, is another measure for AAV-mediated treatment. When AAV was administered at P2, an attenuated level of triglycerides was observed in the liver at the age of six months. Similarly, other parameters, such as cholesterol level in the liver and spleen and triglycerides in the spleen, were reduced when AAV was administered at P120. These results indicate that a large amount of vector (8.4 × 10^13^ vg/kg) was required for the best therapeutic effect in the mouse model. Notably, hepatic inflammation was also attenuated when the AAV vector was administered in a dose-dependent manner at a later stage.

## 7. Future Perspectives

As mentioned earlier, LAL-D is a rare disorder with several treatment options. The prevalence of LAL-D may vary depending on the ethnicity and disease population. The outcome of high-risk FH screening demonstrated that this population contains a large number of LAL-D-affected individuals. To implement screening for LAL-affected individuals, an estimation of the prevalence is required.

## Figures and Tables

**Table 1:** The *LIPA* gene mutations.

Study	Year	Country	Author	Population	n Total	Children	Adults	E8SJM c.G894A		Method	Ref.
								Homo Zygote	Hetero Zygote		
High-risk screening	2022	Slovenia	Suster, U	FH	669	669	0	3	0	NGS/Sanger	[20]
	2021	Italy	Pasta, A	Hypoapolipoproteinemia or mixed hyperlipemia	74	0	74	NR	NR	Sanger	[21]
	2021	Russia	Miroshnikova, VV	Suspected FH	59	28	31	1	0	NGS/Sanger	[22]
	2019	U.K.	Ashfield-Watt, P	FH with no mutation in LDLR, ApoB, and Pcsk9.	663	0	663	0	3	Sanger+TaqMan	[23]
	2016	Netherlands	Sjouke, B	FH with no mutation in LDLR and ApoB	276	63	213	0	2	Sanger	[24]
	2015	U.S.	Pullinger, CR	Dyslipidemia	1357	Combined		0	6	Sanger	[25]
	2013	Germany	Muntoni, S	Selected cohorts	2023	Not described		0	10	Sanger	[26]
	2007	Germany	Muntoni, S	Selected cohorts	2023	Not described		0	10	Allele-selective PCR	[27]
Confirmed cases	2017	Italy	Pisciotta, L	LAL-D-confirmed patients	16	14	2	4	5	Sanger	[28]

FH, Familial Hypercholesterolemia.

**Table 4:** Phenotype of *Lipa*-deficient mice.

Category	Genotype	LAL Activity	Phenotype	Year	Ref.
Global KO	*Lipa*(-/-)	Deficient	↑CE	1998	[65]
	*Lipa*(-/-)	Deficient	↑CE; →TG	2001	[66]
	*Lipa*(-/-)	Deficient	→TG	2016	[67]
	*Lipa*(-/-)	Deficient	T cell defect	2009	[68]
	*Lipa*(-/-)	Deficient	Anemia; ↑Sca-1(+)c-kit(+); ↑GMP	2010	[69]
	*Lipa*(-/-)	Deficient	↑MDSC	2014	[70]
	*Lipa*(-/-)	Deficient	↑T- and B-regulatory cell homeostasis	2021	[71]
	*Lipa*(-/-)	Deficient	↑MDSC function	2022	[72]
	*Lipa*(-/-)	Deficient	↑EC transmigration; ↓Apoptosis; ↑Angiogenesis; ↑Tube formation.	2014	[70]
	*Lipa*(-/-)	Deficient	↑Endothelial Rab7	2017	[73]
	*Lipa*(-/-)	Deficient	↓Osteoblastogenesis	2021	[74]
Conditional KO (cKO)	Alb-Cre; cKO	Liver-specific deficient	↑Hepatic inflammation; ↓Diet-induced obesity	2019	[75]
	Alb-Cre; cKO	Liver-specific deficient	↑CE; →TG; →RA (normal chow); ↑CE; ↓TG; ↓RA (VitA/HFD)	2019	[64]
Tg/KO	c-fms-tg/*Lipa*(-/-)	Myeloid-specific expression	Normalized MDSC function	2011	[76]
	Lap-tg/*Lipa*(-/-)	Liver-specific expression	Normalized melanoma metastasis	2015	[77]
	CCSP-tg/*Lipa*(-/-)	Lung alveolar type II epithelial cell-specific expression	Normalized tumor metastasis;Normalized Lung inflammation	2016	[78]

Alb, albumin; CCSP, Clara cell secretory protein; cKO, conditional knockout; GMP, *Guanosine* monophosphate; HFD, high-fat diet; Lap, liver-activated promoter; MDSC, myeloid-derived suppressor cells; Tg, transgenic; VitA, vitamin A; ↑, increase; ↓, decrease; →, no change.

**Table 5:** Preclinical studies.

Method	Year	Enzyme/Vector	Dose	Route	Ref.
ERT	2005	phLAL, chLAL	3.95 mg/kg	Intraperitoneal	[88]
	2008	hLAL (Nicotiana benthamiana)	1.65–6.65 mg/kg	Intraperitoneal	[89]
	2014	hLAL	0.8–10 mg/kg	Intravenous	[86]
GT	2003	Adenovirus	3 × 10^8^ PFU	Intravenous	[90]
	2021	AAV8	5 × 10^11^ vc/mouse	Intravenous	[85]
	2022	scAAVrh74	8.4 × 10^13^ vg/kg	Intravenous	[91]

AAV, adeno-associated virus; ch, Chinese hamster ovary; ERT, enzyme replacement therapy; GT, gene therapy; p, Pichia pastoris.

## Data Availability

Not applicable.
